# Concurrent Preoperative Presence of Hydronephrosis and Flank Pain Independently Predicts Worse Outcome of Upper Tract Urothelial Carcinoma

**DOI:** 10.1371/journal.pone.0139624

**Published:** 2015-10-15

**Authors:** Hsin-Chih Yeh, Hau-Chern Jan, Wen-Jeng Wu, Ching-Chia Li, Wei-Ming Li, Hung-Lung Ke, Shu-Pin Huang, Chia-Chu Liu, Yung-Chin Lee, Sheau-Fang Yang, Peir-In Liang, Chun-Nung Huang

**Affiliations:** 1 Department of Urology, Kaohsiung Municipal Ta-Tung Hospital, Kaohsiung, Taiwan; 2 Department of Urology, Kaohsiung Medical University Hospital, Kaohsiung, Taiwan; 3 Department of Urology, School of Medicine, College of Medicine, Kaohsiung Medical University, Kaohsiung, Taiwan; 4 Graduate Institute of Medicine, College of Medicine, Kaohsiung Medical University, Kaohsiung, Taiwan; 5 School of Post-Baccalaureate Medicine, College of Medicine, Kaohsiung Medical University, Kaohsiung, Taiwan; 6 Center for Infectious Disease and Cancer Research, Kaohsiung Medical University, Kaohsiung, Taiwan; 7 Center for Stem Cell Research, Kaohsiung Medical University, Kaohsiung, Taiwan; 8 Department of Urology, Ministry of Health and Welfare Pingtung Hospital, Pingtung, Taiwan; 9 Department of Pathology, Kaohsiung Municipal Ta-Tung Hospital, Kaohsiung, Taiwan; 10 Department of Pathology, Kaohsiung Medical University Hospital, Kaohsiung, Taiwan; 11 Department of Pathology, School of Medicine, College of Medicine, Kaohsiung Medical University, Kaohsiung, Taiwan; Hunter College of The City University of New York, UNITED STATES

## Abstract

**Objectives:**

To investigate the impact of preoperative hydronephrosis and flank pain on prognosis of patients with upper tract urothelial carcinoma.

**Methods:**

In total, 472 patients with upper tract urothelial carcinoma managed by radical nephroureterectomy were included from Kaohsiung Medical University Hospital Healthcare System. Clinicopathological data were collected retrospectively for analysis. The significance of hydronephrosis, especially when combined with flank pain, and other relevant factors on overall and cancer-specific survival were evaluated.

**Results:**

Of the 472 patients, 292 (62%) had preoperative hydronephrosis and 121 (26%) presented with flank pain. Preoperative hydronephrosis was significantly associated with age, hematuria, flank pain, tumor location, and pathological tumor stage. Concurrent presence of hydronephrosis and flank pain was a significant predictor of non-organ-confined disease (multivariate-adjusted hazard ratio = 2.10, *P* = 0.025). Kaplan-Meier analysis showed significantly poorer overall and cancer-specific survival in patients with preoperative hydronephrosis (*P* = 0.005 and *P* = 0.026, respectively) and in patients with flank pain (*P* **<** 0.001 and *P* = 0.001, respectively) than those without. However, only simultaneous hydronephrosis and flank pain independently predicted adverse outcome (hazard ratio = 1.98, *P* **=** 0.016 for overall survival and hazard ratio = 1.87, *P* = 0.036 for and cancer-specific survival, respectively) in multivariate Cox proportional hazards models. In addition, concurrent presence of hydronephrosis and flank pain was also significantly predictive of worse survival in patient with high grade or muscle-invasive disease. Notably, there was no difference in survival between patients with hydronephrosis but devoid of flank pain and those without hydronephrosis.

**Conclusion:**

Concurrent preoperative presence of hydronephrosis and flank pain predicted non-organ-confined status of upper tract urothelial carcinoma. When accompanied with flank pain, hydronephrosis represented an independent predictor for worse outcome in patients with upper tract urothelial carcinoma.

## Introduction

Upper tract urothelial carcinoma (UTUC), including tumor from urothelium of renal pelvis and ureter, is a rare malignancy. UTUC accounts for approximately 5–10% of all urothelial tumors, with an estimated incidence of 2.08 cases per 100,000 person-years in the United States [1], while the incidence of UTUC in Taiwan is as high as 30% of all urothelial carcinomas [2,3], which is obviously higher than that worldwide. The gold standard management for UTUC is radical nephroureterectomy due to its high recurrence rate in the remaining upper tract [4]. Additional regional lymphadenectomy and neoadjuvant chemotherapy should be considered in patients with high risk disease. Conversely, more conservative approaches may be applied in patients with low risk disease, such as endoscopic ablation or segmental resection [4–6].

Several powerful prognostic factors for UTUC have been identified, including pathological tumor stage (pT), tumor grade, lymph node (LN) involvement, and lymphovascular invasion. These factors predict disease outcome and therefore are helpful in providing proper therapeutic strategy for patients with UTUC according to their risk of progression. However, most of them were obtained postoperatively [7]. Prognostic factors that are routinely acquired in the preoperative setting, like hydronephrosis and flank pain, can even be more valuable since they afford additional information before surgery. Patients with adverse preoperative features may benefit from neoadjuvant chemotherapy when both renal units are in place [8].

Previous studies have confirmed the concept that preoperative hydronephrosis is predictive for advanced pT in UTUC [9–13]. With regard to survival, many studies have proven the negative impact of preoperative hydronephrosis in univariate analyses [14–17]. However, results of preoperative hydronephrosis as an independent prognostic factor of UTUC in multivariate analyses were discrepant [14,15,17]. Therefore, further evaluation of the role of hydronephrosis in UTUC is certainly required. In addition, although Cho et al. reported that grade of hydronephrosis is correlated with pT in patients with ureteral cancer [18], the ensuing studies have demonstrated that high grade hydronephrosis alone is not a reliable factor for disease progression and outcome in either ureteral cancer or UTUC [10,11,19,20].

Clinically, hydronephrosis and flank pain are highly correlated. Sudden occurrence of hydronephrosis can result in intense pain in the flank area. In contrast, gradual development of hydronephrosis will generally cause dull pain with no attacks of renal colic. Ataus et al. noticed that UTUC patients presenting with flank pain had significantly poorer outcome compared to those with hematuria or bladder tumor [21]. Besides, Inman et al. observed that the presence of constitutional symptoms such as flank pain or weight loss was associated with poor overall survival (OS) in patients with UTUC [22].

Flank pain in UTUC is believed to be secondary to hydronephrotic distension or related to local tumor mass effect. In previous studies, preoperative hydronephrosis has never been combined with related symptoms to evaluate outcome of UTUC. Therefore, in this retrospective study, we aimed to investigate this issue by analyzing the influence of preoperative hydronephrosis and flank pain at the same time on predicting non-organ-confined (NOC) status, OS, and cancer-specific survival (CSS) of UTUC.

## Materials and Methods

This study included 472 patients who underwent radical nephroureterectomy for UTUC at Kaohsiung Medical University Hospital Healthcare System between 1991 and 2013. This research was approved by the Institute Review Board of the Kaohsiung Medical University Hospital (KMUH-IRB-20120138). All patient records were anonymized and de-identified prior to analysis. Patients were not recruited into this study if they did not receive radical surgery, had evidence of metastasis at diagnosis, or died within 14 days after surgery. Dilatation of renal pelvis and calyx was determined by renal ultrasonography, computed tomography, or intravenous pyelography. As previously described, any degree of renal collecting dilatation was defined as hydronephrosis [23]. We excluded patients whose hydronephrosis was not caused by tumor, such as urolithiasis or stricture. The intensity of flank pain was determined by verbal descriptor scale, i.e. no, slight, mild, moderate, and severe pain. Patients were regarded to have cancer-related flank pain if the pain was moderate to severe and developed at the same side of UTUC based on their own complaints from medical record reviews. Those with mild pain or ill-defined, nonspecific lumbago were considered to have no flank pain. Tumors were staged according to AJCC TNM classification, 7^th^ edition and graded using the 2004 WHO/International Society of Urologic Pathology consensus classification. All cases formerly graded by three-grade WHO system were reviewed by two pathologists and were re-classified as low or high grade according to the 2004 WHO classification. In brief, low grade tumors are evenly distributed and have uniform nuclei, mild increase of nuclear-to-cytoplasmic ratio, fine chromatin, and rare mitoses. In contrast, high grade tumors tend to show irregular distribution of tumor cells, presence of pleomorphic nuclei, coarse chromatin, and frequent mitoses. Other clinicopathological parameters were also recorded for analysis, including age, gender, smoking, preoperative estimated glomerular filtration rate (eGFR), surgery method, tumor location (renal pelvis, ureter, or synchronous), pT, tumor grade, LN involvement, and adjuvant chemotherapy. eGFR was calculated with the MDRD study equation: 186 × (serum creatinine)^-1.154^ × (age)^-0.203^ × (0.742 if female) [24]. Patients were considered to have chronic kidney disease (CKD) if they had an eGFR < 60 ml/min/1.73m^2^ or received regular dialysis. Regular cystoscopy, urine cytology and periodic imaging studies were mandated postoperatively in every patient following institutional guideline. In the first 2 years after radical surgery, patients were followed every 3 months, then every 6 months in the next 2 years, and annually thereafter.

Demographic and clinicopathological factors were categorized and compared between patients with and without preoperative hydronephrosis using chi-square test. Kaplan–Meier method was applied to analyze OS and CSS, and the significant differences of which were determined by the log-rank test. Preoperative parameters and potential prognostic factors were enrolled into univariate and multivariate Cox proportional hazards models to identify predictors of NOC disease (≥ pT3 and/or positive LN) and survival, respectively. Since hydronephrosis and flank pain were closely related, we divided patients into four groups (those without hydronephrosis and flank pain, those with flank pain but no hydronephrosis, those with hydronephrosis but free from flank pain, and those with both hydronephrosis and flank pain) to realize the respective effect of hydronephrosis and flank pain on prognosis. SPSS software program (version 19.0; SPSS Inc, Chicago, IL, USA) was used for statistical tests. All *P* values were two-sided, and < 0.05 was considered significant.

## Results

### Patients’ Clinicopathological Characteristics

The clinicopathological characteristics of 472 patients were listed in Table 1. There were 204 males and 268 females, with a median age of 67 years. The mean (median; SD) follow-up was 48.6 (33; 46.4) months (range: 1–233). In this cohort, 121 (26%) and 399 (85%) patients presented with flank pain and hematuria, respectively. Radical surgery was performed with open method in 269 (57%), while the remaining was operated laparoscopically. Three hundred and sixteen (67%) had an eGFR less than 60 (ml/min/1.73m^2^) and 87 (18%) received adjuvant chemotherapy. One hundred and eighty-nine (40%) tumors were located in the renal pelvis, 193 (41%) in the ureter, and 90 (19%) in both locations. As for pathological features, the stage distribution of UTUC was pTa/Tis, pT1, pT2, pT3, and pT4 in 60 (13%), 130 (27%), 112 (24%), 142 (30%), and 28 (6%) respectively. Forty-one (9%) patients had LN metastasis, and 360 (76%) had high grade tumor. UTUC patients were divided into two groups based on the presence of preoperative hydronephrosis (Table 1). Two hundred and ninety-two patients (62%) had hydronephrosis preoperatively, and 180 (38%) did not. Preoperative hydronephrosis was significantly associated with age (*P* = 0.016), hematuria (*P* < 0.001), flank pain (*P* = 0.002), tumor location (*P* < 0.001), and pT (*P* = 0.041).

**Table 1:** Demographics and clinicopathological characteristics of 472 patients with UTUC treated by radical nephroureterectomy.

		Preoperative hydronephrosis		
	All patients	Presence	Absence	*P* value
**Number of patients, no. (%)**	472 (100)	292 (62)	180 (38)	
**Median age, years (range)**	67 (24–95)	66 (25–90)	67 (24–95)	
**Age, no. (%)**				***0*.*016***
≦ 67	242 (51)	137 (47)	105 (58)	
> 67	230 (49)	155 (53)	75 (42)	
**Gender, no. (%)**				*0*.*969*
Males	204 (43)	126 (43)	78 (43)	
Females	268 (57)	166 (57)	102 (57)	
**Smoking, no. (%)**				*0*.*323*
No	373 (79)	235 (80)	138 (71)	
Yes	99 (21)	57 (20)	42 (29)	
**Hematuria, no. (%)**				***< 0*.*001***
No	73 (15)	64 (22)	9 (5)	
Yes	399 (85)	228 (78)	171 (95)	
**Flank pain, no. (%)**				***0*.*002***
No	351 (74)	203 (70)	148 (82)	
Yes	121 (26)	89 (30)	32 (18)	
**Preoperative eGFR (ml/min/1.73m** ^**2**^ **)**				*0*.*087*
≧ 60	156 (33)	88 (30)	68 (38)	
< 60	316 (67)	204 (70)	112 (62)	
**Surgery method, no. (%)**				*0*.*100*
Laparoscopic	203 (43)	117 (40)	86 (48)	
Open	269 (57)	175 (60)	94 (52)	
**Tumor location, no. (%)**				***< 0*.*001***
Renal pelvis	189 (40)	89 (30)	100 (55)	
Ureter	193 (41)	145 (50)	48 (27)	
Synchronous	90 (19)	58 (20)	32 (18)	
**Pathological tumor stage, no (%)**				***0*.*041***
Tis/a	60 (13)	38 (13)	22 (12)	
T1	130 (27)	66 (23)	64 (36)	
T2	112 (24)	73 (25)	39 (22)	
T3	142 (30)	95 (32)	47 (26)	
T4	28 (6)	20 (7)	8 (4)	
**Lymph node status, no. (%)**				*0*.*098*
pN_0_	170 (36)	96 (33)	74 (41)	
pN_X_	261 (55)	166 (57)	95 (53)	
pN_1/2_	41 (9)	30 (10)	11 (6)	
**Pathological tumor grade, no. (%)**				*0*.*104*
Low	112 (24)	62 (21)	50 (28)	
High	360 (76)	230 (79)	130 (72)	
**Adjuvant chemotherapy, no. (%)**				*0*.*656*
No	385 (82)	240 (82)	145 (81)	
Yes	87 (18)	52 (18)	35 (19)	

*eGFR* estimated glomerular filtration rate.

### Predictors of NOC Disease in Preoperative Model

In total, 36.7% (173/472) had NOC disease. Among them, 68% (118/173) had preoperative hydronephrosis and 32% (55/173) had flank pain. In the model to predict NOC disease before surgery, both univariate and multivariate analyses showed that male gender (*P* = 0.019 and 0.045, respectively), synchronous tumor location (*P* = 0.005 and = 0.034, respectively), absence of hematuria (*P* = 0.001 and 0.010, respectively), and simultaneous presence of hydronephrosis and flank pain (*P* = 0.003 and 0.025, respectively) were significant predictors (Table 2).

**Table 2:** Univariate and multivariate analyses for prediction of non-organ confined disease (≥ pT3 and/or positive lymph nodes).

	Non-organ confined disease					
	Univariate analysis			Multivariate analysis		
Variables	HR	95% CI	*P value*	HR	95% CI	*P value*
**Age**						
> 67 vs. ≦ 67	0.954	0.656–1.387	*0*.*804*	0.921	0.617–1.375	*0*.*687*
**Gender**						
Male vs. Female	1.574	1.078–2.296	***0*.*019***	1.604	1.012–2.543	***0*.*045***
**Smoking**						
Yes vs. No	1.223	0.777–1.926	*0*.*384*	1.083	0.620–1.892	*0*.*778*
**Preoperative eGFR (ml/min/1.73m** ^**2**^ **)**						
< 60 vs. ≧ 60	1.141	0.764–1.703	*0*.*519*	1.255	0.818–1.927	*0*.*299*
**Tumor location**			***<0*.*001***			***<0*.*001***
Ureter vs. Renal pelvis	0.693	0.451–1.066	*0*.*095*	0.496	0.310–0.795	***0*.*004***
Synchronous vs. Renal pelvis	2.078	1.249–3.460	***0*.*005***	1.775	1.044–3.018	***0*.*034***
**Hematuria**						
Yes vs. No	0.413	0.249–0.684	***0*.*001***	0.449	0.245–0.823	***0*.*010***
**HN and flank pain**			***0*.*026***			*0*.*144*
Flank pain without HN vs. no HN and flank pain	1.238	0.551–2.784	*0*.*605*	1.190	0.508–2.787	*0*.*690*
HN without flank pain vs. no HN and flank pain	1.356	0.861–2.135	*0*.*189*	1.458	0.899–2.365	*0*.*127*
HN with flank pain vs. no HN and flank pain	2.311	1.340–3.985	***0*.*003***	2.103	1.099–4.025	***0*.*025***

*eGFR* estimated glomerular filtration rate, *HN* hydronephrosis.

### Survival Analysis

**Fig 1:**
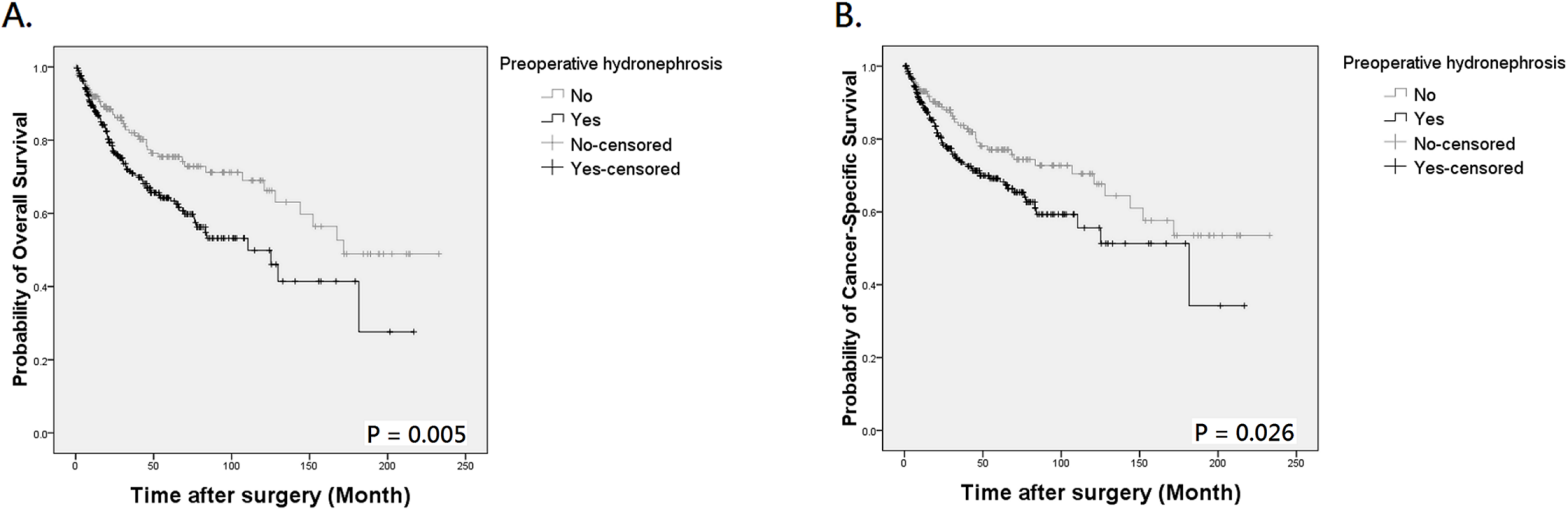
Kaplan-Meier analysis for overall survival (A) and cancer-specific survival (B) in 472 patients with UTUC according to the presence of preoperative hydronephrosis.

**Fig 2:**
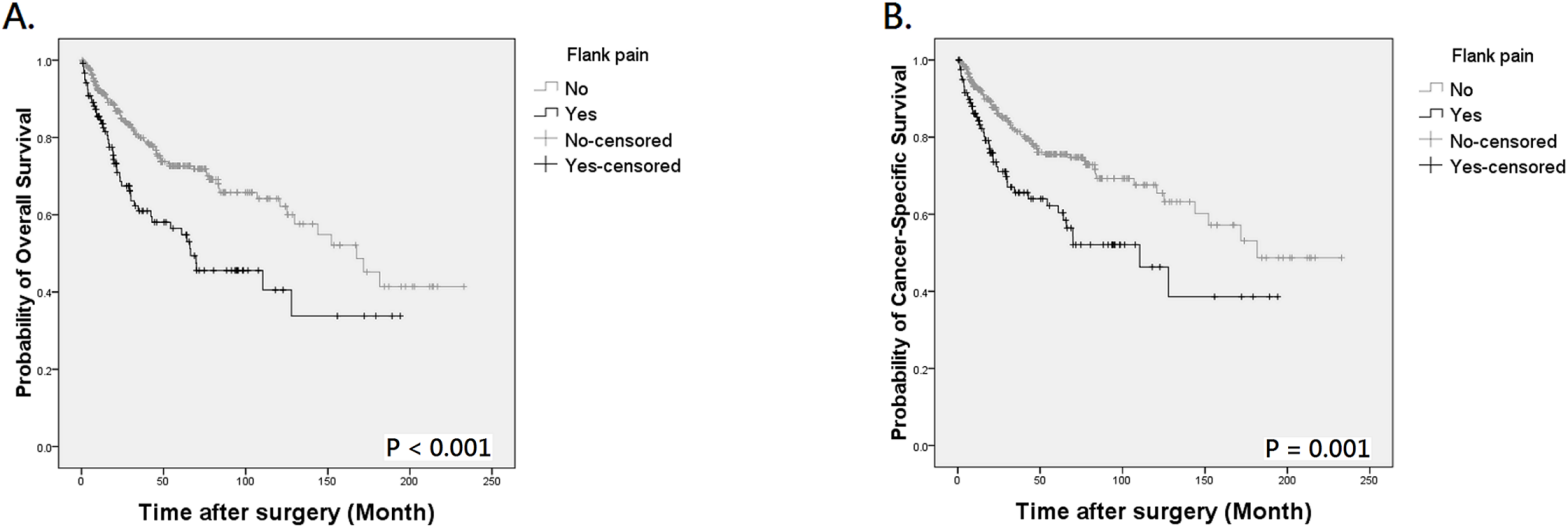
Kaplan-Meier analysis for overall survival (A) and cancer-specific survival (B) in 472 patients with UTUC according to the presence of flank pain.

**Fig 3:**
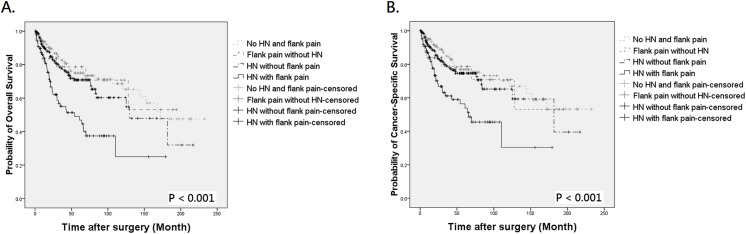
Kaplan-Meier analysis for overall survival (A) and cancer-specific survival (B) in 472 UTUC patients who divided into 4 groups by the presence of preoperative hydronephrosis (HN) and flank pain.

During follow-up, 121 patients died of UTUC, 18 expired due to other causes, and 145 were assessed at 5 years to calculate survival. By Kaplan-Meier method, the 5-year OS and CSS were 64.2% and 69.2% in patients with preoperative hydronephrosis, compared to 75.5% and 77.1% in those without hydronephrosis (Fig 1A, *P* = 0.005 for OS and Fig 1B, *P* = 0.026 for CSS, respectively). The mean OS of patients with and without hydronephrosis was 106.17 and 125.62 months, with a difference of 19.45 months. The difference of mean CSS between the two groups was also clinically meaningful (11.42 months). Similarly, patients with flank pain also had worse outcome than those without (Fig 2A, *P* < 0.001 for OS and Fig 2B, *P* = 0.001 for CSS, respectively). When patients were stratified by the presence of hydronephrosis and/or flank pain, Kaplan-Meier curves indicated an obvious survival decline in patients with concurrent hydronephrosis and flank pain but not in other 3 groups (Fig 3A and 3B, both *P* < 0.001). The differences of mean OS and CSS between those with and without symptomatic hydronephrosis were even more marked (5.48 and 5.40 years, respectively). Interestingly, survival rates were similar among patients with hydronephrosis alone, flank pain alone, and those without hydronephrosis and flank pain.

**Fig 4:**
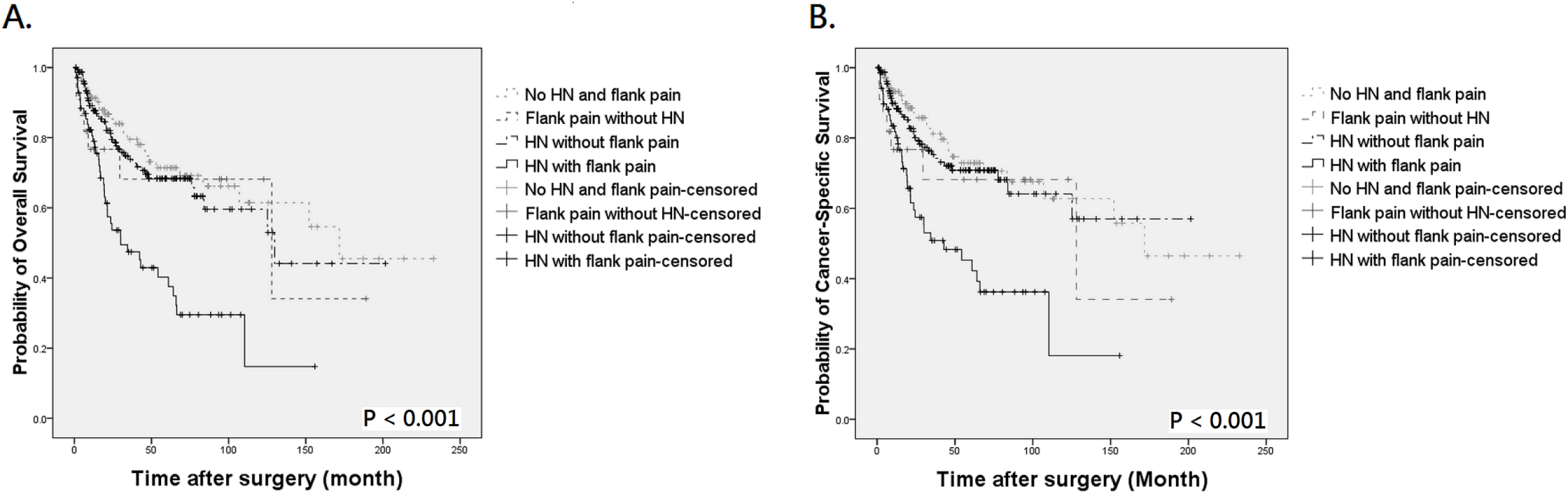
Kaplan-Meier analysis for overall survival (A) and cancer-specific survival (B) in patients with high grade UTUC who divided into 4 groups by the presence of preoperative hydronephrosis (HN) and flank pain.

**Fig 5:**
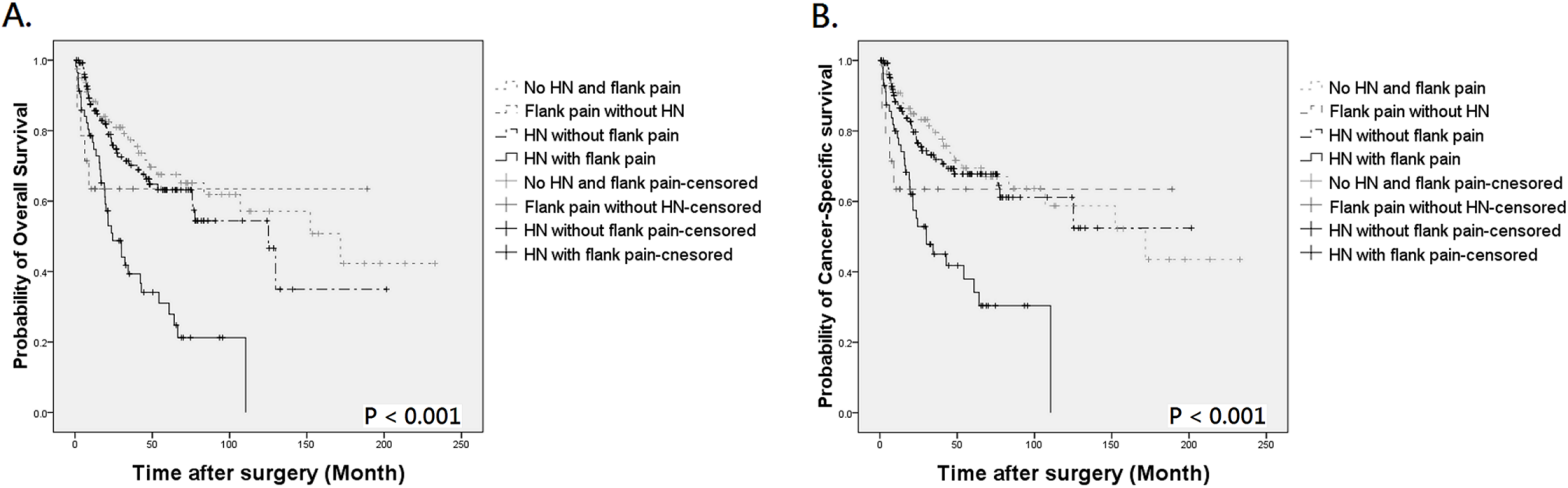
Kaplan-Meier analysis for overall survival (A) and cancer-specific survival (B) in patients with muscle-invasive UTUC who divided into 4 groups by the presence of preoperative hydronephrosis (HN) and flank pain.

As different molecular features may exist in UTUC patients based on tumor grade and stage, we attempted to analyze the effect of symptomatic hydronephrosis on survival between different pathological features. There was no difference in OS and CSS according to the presence of symptomatic hydronephrosis in low grade or non-muscle-invasive UTUC. However, in high grade UTUC, patients with concurrent hydronephrosis and flank pain had a significantly poorer OS and CSS (Fig 4A and 4B, both *P* < 0.001). The adverse outcome was also observed in patients with muscle-invasive disease (Fig 5A and 5B, both *P* < 0.001). These findings indicated that the presence of symptomatic hydronephrosis could identify a subset of patients with worse prognosis in high grade or high stage UTUC.

In Table 3, the univariate analysis revealed that OS was significantly associated with age (*P* = 0.015), preoperative eGFR (*P* = 0.041), tumor location (*P* = 0.032), pT (*P* < 0.001), LN involvement (*P* < 0.001), tumor grade (*P* < 0.001), adjuvant chemotherapy (*P* < 0.001), hematuria (*P* = 0.002), and simultaneous presence of hydronephrosis and flank pain (*P* < 0.001). In multivariate analysis, in addition to aging (*P* = 0.012), poor preoperative eGFR (*P* = 0.030), advanced pT (*P* < 0.001), and LN metastasis (*P* = 0.002), simultaneous presence of hydronephrosis and flank pain was an independent predictor for worse OS (HR = 1.98, 95% CI = 1.137–3.433, *P* = 0.016). The significant factors for CSS in the multivariate analysis were identical to those for OS.

**Table 3:** Univariate and multivariate Cox proportional hazards models predicting overall survival and cancer-specific survival in 472 patients with UTUC.

Age	Overall survival						Cancer-specific Survival					
	Univariate			Multivariate			Univariate			Multivariate		
Variables	HR	95% CI	*P value*	HR	95% CI	*P value*	HR	95% CI	*P value*	HR	95% CI	*P value*
**Age**												
> 67 vs. ≦ 67	1.519	1.083–2.131	***0*.*015***	1.598	1.109–2.304	***0*.*012***	1.595	1.109–2.295	***0*.*012***	1.647	1.111–2.442	***0*.*013***
**Gender**												
Male vs. Female	1.343	0.963–1.874	*0*.*082*	1.238	0.820–1.868	*0*.*310*	1.216	0.850–1.737	*0*.*284*	1.125	0.722–1.753	*0*.*603*
**Smoking**												
Yes vs. No	1.201	0.809–1.784	*0*.*363*	1.295	0.786–2.134	*0*.*309*	1.102	0.714–1.701	*0*.*662*	1.321	0.763–2.289	*0*.*320*
**Preoperative eGFR (ml/min/1.73m** ^**2**^ **)**												
< 60 vs. ≧ 60	1.485	1.017–2.169	***0*.*041***	1.577	1.045–2.382	***0*.*030***	1.699	1.117–2.586	***0*.*013***	1.691	1.071–2.669	***0*.*024***
**Surgery method**												
Laparoscopic vs. Open	0.797	0.557–1.139	*0*.*213*	0.955	0.652–1.399	*0*.*813*	0.784	0.535–1.150	*0*.*214*	0.949	0.630–1.429	*0*.*803*
**Tumor location**			***0*.*032***			*0*.*236*			*0*.*082*			*0*.*268*
Ureter vs. Renal pelvis	1.183	0.803–1.742	*0*.*396*	0.912	0.601–1.384	*0*.*664*	1.002	0.664–1.512	*0*.*993*	0.803	0.515–1.252	*0*.*333*
Synchronous vs. Renal pelvis	1.785	1.149–2.775	***0*.*010***	1.336	0.844–2.117	*0*.*217*	1.605	1.007–2.556	***0*.*047***	1.199	0.737–1.951	*0*.*465*
**Pathological tumor stage**			***<0*.*001***			***<0*.*001***			***<0*.*001***			***<0*.*001***
T1 vs. Tis/a	1.057	0.465–2.401	*0*.*895*	1.061	0.459–2.453	*0*.*890*	1.624	0.606–4.352	*0*.*335*	1.529	0.561–4.168	*0*.*407*
T2 vs. Tis/a	1.924	0.882–4.197	*0*.*100*	2.066	0.880–4.850	*0*.*096*	2.269	0.859–5.993	*0*.*098*	2.171	0.766–6.154	*0*.*145*
T3 vs. Tis/a	4.162	1.991–8.700	***<0*.*001***	3.407	1.458–7.964	***0*.*005***	5.988	2.396–14.964	***<0*.*001***	4.438	1.606–12.266	***0*.*004***
T4 vs. Tis/a	17.146	7.494–39.229	***<0*.*001***	5.767	2.061–16.139	***0*.*001***	27.043	10.079–72.558	***<0*.*001***	8.530	2.634–27.624	***<0*.*001***
**Pathological lymph nodal status**			***<0*.*001***			***0*.*002***			***<0*.*001***			***0*.*005***
pN_x_ vs. pN_0_	1.276	0.858–1.899	*0*.*229*	1.160	0.775–1.735	*0*.*471*	1.274	0.829–1.957	*0*.*269*	1.179	0.763–1.823	*0*.*458*
pN_1/2_ vs. pN_0_	8.334	5.036–13.792	***<0*.*001***	3.109	1.657–5.835	***<0*.*001***	8.791	5.168–14.956	***<0*.*001***	2.943	1.522–5.691	***0*.*001***
**Tumor grade**												
High vs. Low	2.404	1.507–3.835	***<0*.*001***	0.959	0.537–1.715	*0*.*888*	2.983	1.733–5.137	***<0*.*001***	1.202	0.621–2.328	*0*.*586*
**Adjuvant chemotherapy**												
Yes vs. No	2.914	2.048–4.148	***<0*.*001***	1.350	0.884–2.062	*0*.*165*	3.009	2.066–4.381	***<0*.*001***	1.313	0.837–2.058	*0*.*236*
**Hematuria**												
Yes vs. No	0.495	0.317–0.771	***0*.*002***	0.616	0.378–1.003	*0*.*051*	0.477	0.312–0.729	***0*.*001***	0.640	0.380–1.077	*0*.*093*
**HN and flank pain**			***<0*.*001***			*0*.*078*			***0*.*001***			*0*.*130*
Flank pain without HN vs. no HN and flank pain	1.121	0.521–2.413	*0*.*770*	1.561	0.696–3.499	*0*.*280*	1.264	0.582–2.743	*0*.*554*	1.780	0.783–4.049	*0*.*169*
HN without flank pain vs. no HN and flank pain	1.301	0.848–1.996	*0*.*228*	1.146	0.730–1.799	*0*.*553*	1.254	0.795–1.978	*0*.*329*	1.140	0.705–1.842	*0*.*594*
HN with flank pain vs. no HN and flank pain	2.835	1.804–4.454	***<0*.*001***	1.975	1.137–3.433	***0*.*016***	2.607	1.603–4.238	***<0*.*001***	1.874	1.042–3.371	***0*.*036***

*eGFR* estimated glomerular filtration, *HN* hydronephrosis.

## Discussion

UTUC is a rare malignant tumor in the urinary system worldwide. Common clinical presentations of UTUC include hematuria, flank pain, and hydronephrosis. Previous investigations have reported that approximately 70–80% of UTUC patients present with hematuria, 20–40% with flank pain [4,25], and 50–67% with preoperative hydronephrosis [13,15–17,26]. Current strategies for preoperative risk stratification for UTUC include ureteroscopic biopsy, cytology, and modern imaging techniques, but these methods still have limitations. It is important to identify significant prognosticators for better prediction on disease outcome before surgery to facilitate more appropriate individualized treatment. In recent years, a number of studies have been focusing on whether preoperative hydronephrosis has predictive potential for adverse disease features or even survival in patients with UTUC.

The characteristics from this cohort were comparable to other studies concerning mean age, tumor location, and prevalence of preoperative hydronephrosis. Regarding gender distribution in UTUC, our population showed a slight female predominance (male-female ratio = 1:1.31), which was in accordance with other Taiwanese studies but different from those in other countries [3,27]. In this study, 316 (67%) had CKD prior to the diagnosis of UTUC. The fact that over 50% patients with UTUC had CKD was also similar to previous Taiwanese studies [2,27].

In our analysis, we found that preoperative hydronephrosis in patients with UTUC was associated with age, hematuria, flank pain, tumor location and pT. Consistent with previous studies [10,13,14,16,28,29], ureteral tumors had higher prevalence of hydronephrosis than pyelocaliceal tumors (75% vs 47%). Most of the previous studies confirmed the predictive value of hydronephrosis on adverse features of UTUC. Messer et al. reported that preoperative hydronephrosis can predict muscle-invasive, NOC, and high grade UTUC [13]. Results from other studies were similar, showing that hydronephrosis was associated with worse pathological characteristics of UTUC [10,11,18–20]. In multivariate analysis of our study, absence of hematuria, concurrent presence of hydronephrosis and flank pain, tumor location, and male gender were significantly correlated with NOC disease. A recent Chinese study has also demonstrated that males and patients with pyelocaliceal tumors are at higher risk of advanced tumor stage than females and those with ureteral tumors, respectively [12].

However, most studies did not validate the independently prognostic role of hydronephrosis in UTUC. For Sakano et al. and Morizane et al., hydronephrosis was significantly associated with CSS only in univariate analysis but not in multivariate model [15,17]. Ng et al demonstrated that the prognostic effect of hydronephrosis was associated with CSS in the preoperative model, in which only limited confounding variables were adjusted. The significance of hydronephrosis for survival disappeared in the postoperative model after correcting crucial pathological features [11]. In a study by Bozzini et al, preoperative hydronephrosis was not associated with survival in univariate analysis and was predictive for OS and metastasis-free survival merely in the pyelocaliceal tumors [14]. Only one cohort of 217 patients indicated that hydronephrosis was an independent risk factor for CSS in multivariate analysis [16].

There were limited data concerning whether flank pain is associated with oncologic outcomes in patients with UTUC. Only three previous studies evaluated the effect of flank pain on UTUC with small case numbers, and two of them suggested its predictive role on outcome [21,22]. Raman et al. incorporated flank pain, hematuria, and palpable mass as local symptoms of UTUC and found that local symptoms were not associated to prognosis [30]. However, since Ataus et al. has shown that patients with flank pain had worse survival than those with hematuria [21], we believed it is more reasonable to evaluate each symptom separately. Indeed, our results showed conflicting impacts of flank pain and hematuria on prognosis. Presence of flank pain and lack of hematuria may imply advanced disease status, and the marginal protective effect of hematuria on outcome was possibly related to earlier diagnosis.

In our study, preoperative hydronephrosis was significantly correlated with OS and CSS in univariate analysis, but its prognostic impact vanished in multivariate analysis. By taking the presence of flank pain into consideration, we found that concurrent presence of preoperative hydronephrosis and flank pain was significantly associated with OS and CSS in multivariate analysis and denoted a 1.98- and 1.87-fold increase in the risk of overall and cancer-specific death. The independently prognostic effect of symptomatic hydronephrosis is of value in the preoperative setting to achieve better clinical decision making. For example, tumor grade assessed by ureteroscopic biopsy provides important information before radical surgery. According to our results, the presence of symptomatic hydronephrosis can further refine the treatment strategy in patients with high grade UTUC.

Another contribution of this study was that we might explain why there are diversities in the effect of preoperative hydronephrosis on prognosis in previous reports. When patients with preoperative hydronephrosis were dichotomized by the presence of flank pain, the outcome between the two groups was significantly different. As mentioned earlier, patients with symptomatic hydronephrosis did have worse OS and CSS compared to patients without preoperative hydronephrosis or flank pain. However, patients with preoperative hydronephrosis but devoid of flank pain had similar survival as those without hydronephrosis. While no previous study has stratified preoperative hydronephrosis by the presence of flank pain, the effects of hydronephrosis on survival varied consequently.

It has been mentioned that obstruction in the urinary tract is the actual prognostic factor of UTUC [14]. When combined with flank pain, hydronephrosis may be more representative of real obstruction with resultant poor outcome. It was unclear why asymptomatic hydronephrosis would not lead to worse survival than those without hydronephrosis. One possible reason is asymptomatic hydronephrosis may imply that tumor is less aggressive and develops more slowly, thus not causing local flank pain.

There were several limitations in this study. First, the data were collected retrospectively. Second, the patients were treated by different physicians over the period of time. Third, we were unable to evaluate flank pain in exact quantity from patients’ subjective complaints. Fourth, we did not include other preoperative variables which may contribute to risk stratification before surgery, such as urinary cytology, endoscopic findings, or radiographic features. Nevertheless, to our limited knowledge, the effect of preoperative hydronephrosis and flank pain has never been assessed together before. This is the first study to demonstrate its excellent prognostic value, and the novel finding can further assist in individualized treatment strategies in patients with UTUC.

In conclusion, preoperative hydronephrosis and flank pain are useful in predicting advanced UTUC. Compared to preoperative hydronephrosis alone, simultaneous presence of hydronephrosis and flank pain more reliably predicts adverse survival and serve as an independent prognosticator for patients with UTUC.

## Abbreviations

CKD: chronic kidney disease
CSS: cancer-specific survival
eGFR: estimated glomerular filtration rate
LN: lymph node
NOC: non-organ-confined
OS: overall survival
pT: pathological tumor stage
UTUC: upper urinary tract urothelial carcinoma
